# No evidence for LGV transmission among heterosexuals in Amsterdam, the Netherlands

**DOI:** 10.1186/1756-0500-7-355

**Published:** 2014-06-10

**Authors:** Marlies Heiligenberg, Stephan P Verweij, Arjen GCL Speksnijder, Servaas A Morré, Henry JC de Vries, Maarten F Schim van der Loeff

**Affiliations:** 1 Cluster of Infectious Diseases, Public Health Service Amsterdam, P.O. Box 2200, 1000 CE Amsterdam, the Netherlands; 2 Department of Internal Medicine, Division of Infectious Diseases, Center for Infection and Immunity Amsterdam (CINIMA), Academic Medical Center, Meibergdreef 9, 1105 AZ Amsterdam, the Netherlands; 3 Laboratory of Immunogenetics, Department of Medical Microbiology and Infection Control, VU University Medical Center, Amsterdam, the Netherlands; 4 Department of Genetics and Cell Biology, Institute of Public Health Genomics, Research InstituteGROW, Faculty of Health, Medicine and Life Sciences, Maastricht University, 6202 AZ Maastricht, The Netherlands; 5 Department of Dermatology, Academic Medical Center, P.O. Box 22700, 1100 DE Amsterdam, the Netherlands; 6 Centre for Infectious Diseases Control, National Institute for Public Health and the Environment (Rijksinstituut voor Volksgezondheid en Milieu, RIVM), Postbus 13720 BA Bilthoven, the Netherlands; 7 Public Health Service Amsterdam, Nieuwe Achtergracht 100, 1018 WT Amsterdam, the Netherlands

**Keywords:** IgA anti-MOMP, Heterosexual, Lymphogranuloma venereum (LGV), Chlamydia trachomatis

## Abstract

**Background:**

In recent years a few cases of lymphogranuloma venereum (LGV) in heterosexuals in Europe have been reported. It is not known whether LGV transmission among heterosexuals occurs on a wider scale.

**Methods:**

Heterosexual male and female STI clinic clients (n = 587) in Amsterdam, the Netherlands, with a positive nucleic acid amplification test (NAAT) result for Chlamydia trachomatis (CT) were screened for IgA anti-MOMP in serum. If the value was above the cut-off index (2.0) the patient’s CT positive urogenital, ocular or rectal sample(s) were selected and tested for LGV by an in-house LGV-specific NAAT.

**Results:**

Sera of 126 patients were above 2.0 COI. Some patients had >1 CT positive sample. Samples could not be retrieved from 15 of the 126 persons, and 7 samples that were found positive for CT in the diagnostic amplification process could not be confirmed and hence not typed. We did not find a single case of LGV infection in 123 urogenital, ocular or rectal samples from 104 patients.

**Conclusion:**

We found no indications for significant spread of LGV infection in heterosexuals in Amsterdam. Surveillance in females with cervical or anal CT infection is indicated to monitor LGV occurrence in heterosexuals.

## Background

Since 2003, lymphogranuloma venereum (LGV) has been reported in industrialised countries among men who have sex with men (MSM). Most cases are caused by *Chlamydia trachomatis* (CT) biovar L2b, and the majority of the patients are HIV positive [1]. LGV causes an invasive infection, although in the current epidemic a considerable number of patients are asymptomatic or have few symptoms [2]. Through bridging persons (men who have sex with both men and women) the L2b strain could spread from the MSM population to the heterosexual population. In 2009, rectal LGV was diagnosed in a female patient with proctitis in France [3] and in 2011, inguinal LGV was diagnosed in a female patient in the Netherlands, [4] Both women were infected with strain L2b, indicating that in the current LGV epidemic among MSM in Western Europe incidental transmission to heterosexuals occurs. This leads to the question whether these cases of LGV infection in heterosexuals are a needle in a haystack or the tip of an iceberg?

In this retrospective study we attempted to estimate if and to what extent LGV is occurring among heterosexual clients of the sexually transmitted infections (STI) clinic with proven CT infections. The gold standard diagnosis for LGV is based on an in-house developed LGV-specific nucleic acid amplification test (NAAT) [5].

However, LGV-specific NAATs are expensive and laborious, and a screening step would be useful in a population with an expected very low prevalence of LGV. Since LGV is known to cause invasive CT infections, chlamydia serology has been used in the past to support a presumptive LGV diagnosis [6]. Previously we showed that an IgA anti-MOMP (major outer membrane protein) assay had an unexpected high sensitivity and specificity (75.5% and 74.3% respectively) to identify or exclude LGV in CT infected MSM [7]. Although this assay had been evaluated in MSM and not in heterosexuals, we considered it quite likely that LGV in heterosexuals might also lead in the same manner to a positive IgA anti-MOMP result. We used the assay as a screening tool to select patients for LGV-specific NAAT testing. Our goal was not to assess the assay’s sensitivity or specificity in heterosexuals, but to assess if there is an indication of more extensive LGV transmission among heterosexuals in Amsterdam than currently expected.

## Methods

In the STI clinic of the Public Health Service Amsterdam, CT is diagnosed with NAAT (Gen-Probe Aptima Combo 2 Assay, Gen-Probe Incorporated, San Diego, USA). Consecutive heterosexual male and female patients with a urogenital, ocular or rectal CT diagnosis from January until March 2009 were retrospectively selected for this study. Stored sera of these patients were tested for IgA anti-MOMP (*C. trachomatis*-IgA-pELISA Medac, Hamburg, Germany) [7]. The IgA anti-MOMP test results were provided as optical density, calibrated to a positive and negative control sample per microtiter plate and expressed as cut-off index (COI) per sample. If patients had an IgA anti-MOMP assay test result above COI (2.0, the value identified in MSM, as having the highest diagnostic odds ratio for LGV infection[7]), their CT-positive urogenital, ocular or rectal samples, stored at -20ºC, were tested for LGV using the in-house LGV-specific NAAT [8]. The samples were warmed at 45ºC to remove crystals and vortexed until fully dissolved before DNA isolation. The High Pure PCR Template Preparation (HPPTP) Kit (Roche Molecular Biochemicals, Mannheim, Germany) was used to isolate DNA, according to manufacturers’ protocol (version 16.0). DNA was eluted in 2 times 50 μL. LGV detection was performed according to a previously described protocol [8,9]. For this assay, RT-PCR reactions were performed in a volume of 25 μL PCR volume, conditions consisted of TaqMan Mastermix (Applied Biosystems, Foster City, CA, USA), 0.18 μL of each primer, 0.26 μL of probe, and 10 μL prepared sample. Amplification and LGV detection was performed with a LightCycler 480 (Roche Molecular Biochemicals) by standard RT-PCR conditions of the manufacturer, with 45 cycles of 15 seconds at 95°C and 1 minute at 60ºC.

Descriptive statistics were performed using Stata 11 (Stata Corp, College Station, TX, USA). All visitors to the STI clinic are explained that their data and remainders of samples may be used for scientific medical research, after anonymisation of data and samples. Visitors of the clinic who object to such use can indicate this and their data and samples are not used. This approach has been submitted to the Ethics Committee of the Academic Medical Center of the University of Amsterdam, who approved this. Conditions for this approach are that no additional samples are taken and that no additional procedures are conducted. In the research described here only routinely available data and routinely available stored samples were used and the analysis was performed on an anonymised data set. Therefore, specific ethical approval or additional informed consent was needed nor sought.

## Results

Sera from 300 men and 287 women diagnosed with a CT infection were screened for IgA anti-MOMP. Sera of 126 patients were above 2.0 COI. Urogenital, ocular or rectal samples could not be retrieved from 15 of the 126 persons. Demographics, STI history and symptoms, and sexual behaviour in the preceding 6 months of the remaining 111 patients (58 men and 53 women) are described in the Table 1. Median age was 25 years, interquartile range 21–29. Nine patients (8.1%) were coinfected with *Neisseria gonorrhoeae* and 1 patient (0.9%) was coinfected with *T. pallidum* (stage: recent infection). All patients were HIV negative. Many female participants reported having had 2 or more casual partners in the last 6 months (59.5%). Fourteen patients (13.1%) reported having had unsafe anal sex (defined as not always using a condom) in the preceding 6 months. Stored samples from various anatomical regions were tested for LGV. Some patients had more than one sample; in total there were 130 samples from 111 patients: 117 urogenital, 9 rectal, and 4 ocular samples. Seven out of 130 samples that were found positive for CT in the diagnostic amplification process could not be typed in the LGV-specific NAAT. All other samples (n = 123) from 104 patients were negative in the LGV-specific NAAT.

**Table 1:** **Demographic characteristics and sexual behaviour of 111 heterosexual patients with confirmed *Chlamydia trachomatis *** **infection and IgA anti-MOMP serology > 2.0 COI, STI clinic of the Public Health Service Amsterdam, the Netherlands, 2009**

	**Men n (%)**	**Women n (%)**	**Total n (%)**
**Variable**	**N = 58**	**N = 53**	**N = 111**
**Demographics**			
Age categories			
15-19 years	1 (1.7)	14 (26.4)	15 (13.5)
20-24 years	15 (25.9)	25 (47.2)	40 (36.0)
25-29 years	21 (36.2)	10 (18.9)	31 (27.9)
30 or more	21 (36.2)	4 (7.5)	25 (22.5)
Nationality			
Dutch	49 (84.5)	49 (92.5)	98 (88.3)
Other	9 (15.5)	4 (7.5)	13 (11.7)
**STI**			
Location of Ct infection(s)*			
Urogenital	58 (100)	53 (100)	111 (100)
Rectal	0	9 (17.0)	9 (8.1)
Ocular	2 (3.4)	2 (3.8)	4 (3.6)
Bacterial coinfections			
Gonorrhoea	4 (6.9)	5 (9.4)	9 (8.1)
Infectious syphilis	1 (1.7)	0	1 (0.9)
**STI history and symptoms**			
History of syphilis, Ct, or gonorrhoea			
No	58 (100.0)	46 (86.8)	104 (93.7)
Yes	0	7 (13.2)	7 (6.6)
STI symptoms at current visit			
No	25 (43.1)	37 (69.8)	62 (55.9)
Yes	33 (56.9)	16 (30.2)	49 (44.1)
Notified by sexual partner with confirmed STI			
No	41 (70.7)	36 (68.0)	77 (69.4)
Yes	17 (29.3)	17 (32.1)	34 (30.6)
**Sexual behaviour**			
Number of sexual partners in preceding 6 mo.^†^			
0		1 (1.9)	
1		17 (32.1)	
2		23 (43.4)	
3 or more		12 (22.6)	
Anal sex in last 6 mo.‡			
None	49 (89.1)	43 (82.7)	92 (86.0)
Always with a condom	0	1 (1.9)	1 (0.9)
Not always with a condom	6 (10.9)	8 (15.4)	14 (13.1)

*Abbreviations:STI* sexually transmitted infections, *anti-MOMP* anti-major outer membrane protein, Ct  *Chlamydia trachomatis*, *mo.* months, *COI*  cut-off index. * = as some patients had multiple *CT* -infections at more than one anatomical site, totals exceed 100% † = the clinic protocol for heterosexual men does not include this question ‡ = data missing for 3 men and 1 woman.

## Discussion

Following recent case reports of heterosexuals infected with the LGV L2b strain, we studied whether LGV is occurring among the heterosexual population in Amsterdam. Among over hundred heterosexual STI clinic clients with a proven CT infection and a high IgA titer, not a single case of LGV was detected. This suggests that LGV is very rare among heterosexuals in Amsterdam.

Various studies have described prevalence of LGV in MSM, [2,10-12] but information about prevalence of LGV in heterosexual men and women in industrialized countries is mostly limited to female patients diagnosed with rectal CT or from samples collected before 2006. Three small studies in rectal CT-positive heterosexual women found no LGV: studies in 2003–2007 in the USA (n = 31), in 2006–2008 in the UK (n = 20), and in 2001–2005 in the Netherlands (n = 63) [13-15]. In a reply to an article it was mentioned that no LGV was found in urethral or cervical samples from 1567 females and 1095 men collected between 2004 and 2008 in France, however details of this study are lacking and this study has never been formally published [16]. Finally, no LGV was found in 721 CT positive urogenital specimens from men and women collected in private general practices in Switzerland between 2004 and 2006 [17] . In recent years, sporadic cases of heterosexual LGV were diagnosed in symptomatic patients in Europe: LGV (non-L2b) serovars were found in 4 women and 2 men in Portugal and a couple in Spain [18,19]. Two recent case-reports each describe one woman with an LGV strain L2b [3,4].

A limitation of our study is that we did not test all consecutive CT positive heterosexual clients of the STI clinic for LGV infection. Since the serology test was already evaluated in MSM we used it as a screening tool to identify possible LGV cases among heterosexuals. We reasoned that if CT positive individuals would have an LGV infection, these individuals would be more likely to have higher IgA anti-MOMP titers than low titers, similar to MSM. We did not find any LGV infections in those with high titers suggesting LGV is very rare in this population. It is possible that IgA anti-MOMP titres were not yet above COI in presymptomatic infections; therefore, we might have missed LGV cases. Another limitation of our study is that the IgA anti-MOMP assay and the COI value >2.0 have been validated in an MSM population and not in heterosexuals. Given that there have only been a few reported LGV cases in heterosexuals in Europe, the assay cannot be validated in heterosexuals. Another limitation was that we were unable to type 7 of the 130 for LGV; this was not unexpected, as the Aptima Combo 2 assay (used for regular CT diagnosis in the clinic) is much more sensitive, than the LGV-NAAT [20].

A strength of this study is that we included heterosexual men in this study, in contrast to most other studies which either did not include heterosexual men, or did not specify the sexual orientation of the men included. Second, while the only other well-documented larger study analysed samples from 2004 to 2006, [17] the samples tested in our study were collected in 2009, several years after the start of the epidemic among MSM and after the first case reports of heterosexuals with LGV. Substantial spread to the heterosexual population could have occurred in these years. Third, we chose to select on IgA anti-MOMP titres above COI, in stead of selecting samples from symptomatic patients for LGV testing, as LGV may be asymptomatic [2].

## Conclusion

In conclusion, samples from 104 heterosexual male and female CT patients, selected based on their high IgA anti-MOMP results, were negative for LGV. Our findings suggest that currently there is no evidence for significant LGV spread from the MSM population to the heterosexual population in Amsterdam, the Netherlands. Therefore, it seems more likely that the reported LGV cases in heterosexuals are needles in a haystack rather than the top of an iceberg. It seems indicated to conduct surveillance of LGV among heterosexuals. The best approach might be to test for LGV in female clients of STI clinics with confirmed cervical or anal CT infection, as this groups would be the first to be infected.

## Competing interests

The authors declare that they have no competing interests.

## Authors’ contributions

MH, TH and MSVDL designed the study. SV, AS and SM were responsible for the laboratory work and data analysis. MH and MSVDL were responsible for data management. MH, HdV, SM and MSVDL wrote the paper. All authors assisted in revising the manuscript. All authors have seen and approved the final, submitted version of the manuscript.
